# Novel system uses probasin-based promoter, transcriptional silencers and amplification loop to induce high-level prostate expression

**DOI:** 10.1186/1472-6750-7-9

**Published:** 2007-02-12

**Authors:** Jan Woraratanadharm, Semyon Rubinchik, Hong Yu, John Y Dong

**Affiliations:** 1 Department of Microbiology and Immunology, Medical University of South Carolina, Charleston, South Carolina, USA; 2 Division of Cancer Therapeutics, GenPhar Inc., Mount Pleasant, South Carolina, USA

## Abstract

**Background:**

Despite several effective treatment options available for prostate cancer, it remains the second leading cause of cancer death in American men. Thus, there is a great need for new treatments to improve outcomes. One such strategy is to eliminate cancer through the expression of cytotoxic genes specifically in prostate cells by gene therapy vectored delivery. To prevent systemic toxicity, tissue- and/or cancer-specific gene expression is required. However, the use of tissue- or cancer-specific promoters to target transgene expression has been hampered by their weak activity.

**Results:**

To address this issue, we have developed a regulation strategy that includes feedback amplification of gene expression along with a differentially suppressible tetracycline regulated expression system (DiSTRES). By differentially suppressing expression of the tetracycline-regulated transcriptional activator (tTA) and silencer (tTS) genes based on the cell origin, this leads to the activation and silencing of the TRE promoter, respectively. *In vitro *transduction of LNCaP cells with Ad/*GFP*_DiSTRES _lead to GFP expression levels that were over 30-fold higher than Ad/CMV-*GFP*. Furthermore, Ad/FasL-GFP_DiSTRES _demonstrated cytotoxic effects in prostate cancer cells known to be resistant to Fas-mediated apoptosis.

**Conclusion:**

Prostate-specific regulation from the DiSTRES system, therefore, serves as a promising new regulation strategy for future applications in the field of cancer gene therapy and gene therapy as a whole.

## Background

While the prostate cancer death rate has been decreasing steadily in the U.S. since the 1990s, prostate cancer remains the second leading cause of cancer death in men, with an estimated 27,050 deaths predicted to occur in 2007 [1]. When the disease is confined to the prostate, it can be cured by radical prostatectomy or irradiation therapy. However, there are no curative therapies for locally advanced, recurrent, or metastatic diseases. There is, therefore, a need to investigate alternative treatment strategies to improve these outcomes. One such approach is the use of cytotoxic gene therapy vectors directed against prostate cancer cells.

Several prostate cancer gene therapy strategies have been or are currently being evaluated in clinical trials [2]. These treatments have been well tolerated and have shown some indications of biological activity. In 2004 and 2005, the Chinese State Food and Drug Administration (SFDA) approved the first gene therapy-based products for cancer treatment: Gendicine (Sibiono GeneTech Co.) and H101 (Sunway Biotech Co.) [3,4]. The effectiveness of these two adenovirus-based products are quite encouraging for similar vectors being developed in the US, Advexin (Introgen) and ONYX-015 (Onyx Pharmaceuticals).

The ideal vector for prostate cancer gene therapy would be one that is both prostate-specific yet still elicits highly induced expression of the therapeutic gene. Previously, we developed and characterized a novel gene therapy regulation system known as the positive feedback loop with prostate specificity, or PFLPS, regulation system [5]. This system initiated a positive feedback loop of gene expression specifically in cells of prostate origin while retaining prostate-specificity even at MOI as high as 1000 [5]. The PFLPS system was developed by making specific modifications of the Tet-off regulation system (originally developed by Dr. Hermann Bujard [6]) to include the prostate-specific ARR2PB promoter (originally developed by Dr. Robert J. Matusik [7]).

While the PFLPS regulation system worked well for inducing GFP expression, incorporation of the pro-apoptotic FasL-GFP fusion into the PFLPS system gave only modest levels of cytotoxicity (Woraratanadharm, unpublished). In an attempt to improve the cytotoxicity of our vector, we developed an alternative strategy for achieving amplified prostate-specific transcriptional regulation. This system, which we have named the differentially suppressible tetracycline regulated expression system, or DiSTRES, was incorporated into a replication-incompetent Ad vector deleted in E1, E3, and E4 (except for E4orf6), and is similar to PFLPS, as it incorporates positive feedback loop amplification of gene expression as a result of incorporation of the TRE-ARR2PB promoter. However, the DiSTRES regulation strategy differs from PFLPS in that it investigates an alternative mechanism for regulating gene expression by including additional transcriptional regulatory elements to differentially suppress transgene expression depending on the cell type transduced.

In this study, we describe the development and characterization of our new DiSTRES regulation system. The DiSTRES system demonstrated highly induced, prostate-specific gene expression when incorporated into a complex Ad vector. In the presence of dihydrotestosterone (DHT), these levels could be induced to levels up to 30 times higher than that of the CMV promoter and up to 10 times higher than our non-specific tet-regulated control. In addition, when FasL-GFP expression was mediated by the DiSTRES system, this vector demonstrated prostate-specific cytotoxicity, even in the FasL-resistant LNCaP cell line. Prostate-specific regulation from the DiSTRES system, therefore, serves as a promising new regulation strategy for future applications in the field of cancer gene therapy and gene therapy as a whole.

## Results

### Design of the Differentially Suppressible Tetracycline Regulated Expression System (DiSTRES)

The Tet-off system was originally designed by Hermann Bujard's group [6]. In this system, the tetracycline-regulated transcriptional activator protein (tTA) was developed to have binding affinity for a tandem repeat of DNA sequences known collectively as the tetracycline-responsive element (TRE). Binding of tTA to the TRE causes transcriptional activation of downstream promoters. The addition of tetracycline or its analog, doxycycline, to the system causes the tTA to lose its affinity for the TRE, and therefore, results in a loss of transcriptional activation.

Bujard's group also developed an opposing system, which utilizes the tetracycline-regulated transcriptional silencer protein (tTS) [8]. Like tTA, tTS binds to the TRE and loses its affinity for the TRE in the presence of tetracycline or doxycycline. Opposite from tTA, however, binding of tTS to the TRE leads to transcriptional *silencing *(not activation) of downstream promoters. Therefore, differential expression of tTA in certain contexts and tTS in other contexts can be utilized to either turn "on" or "off" a particular gene's expression, respectively.

**Figure 1:**
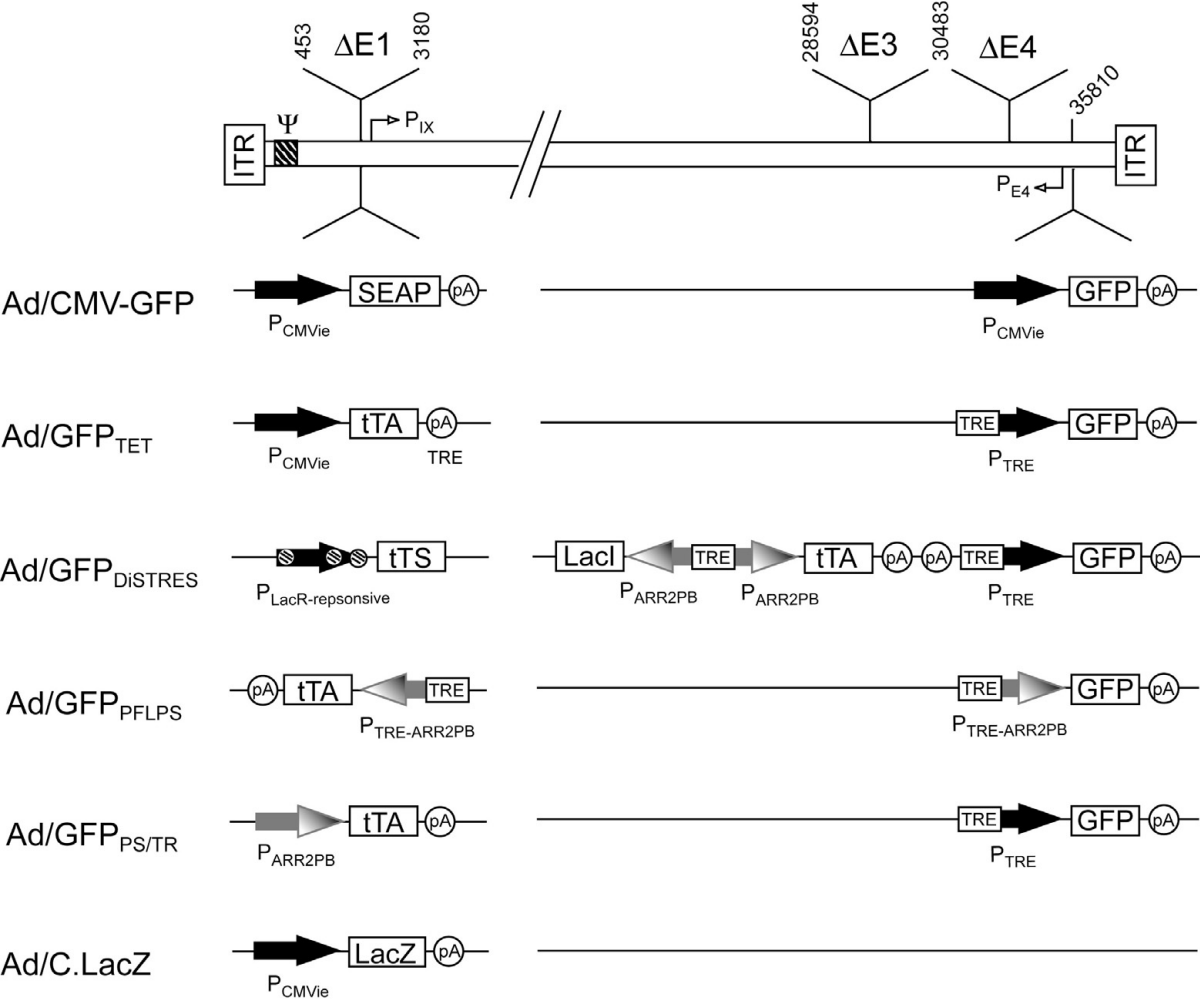
**Structure of the Ad/GFP_DiSTRES _vector and controls**. All transgene cassettes were first cloned into pLAd and pRAd shuttle vectors as described in the Methods section. Vector genomes were then assembled *in vitro*, as described previously [30, 31]. The resulting rAd vectors are E1-deleted and have a deletion in the E3 region (E3 promoter is retained). They also lack all of the E4 ORFs, except orf6, which is expressed from the E4 promoter.

Our first generation Tet-regulated, prostate-specific (PS/TR) vector [9] was developed by a slight modification of the Tet-off system in which the tTA was driven by the prostate-specific ARR2PB promoter instead of by the CMV promoter (see Fig. 1). This vector lacked the positive feedback loop incorporated into both the PFLPS and DiSTRES vectors. It also demonstrated a low level of detectable transgene expression in non-prostate cells. We believed this to be the result of a slight induction of the TRE promoter even in the absence of tTA expression. To improve this system, we developed the PFLPS system.

In the PFLPS regulation system, we cloned the TRE upstream of the ARR2PB promoter to develop a prostate-specific, tTA-inducible promoter known as the TRE-ARR2PB promoter (see Fig. 1). Using the TRE-ARR2PB promoter to drive the expression of both tTA and GFP led to a positive feedback loop which was only activated in prostate cells [5]. This activity was effective at inducing GFP expression, but FasL-GFP induction was somewhat modest.

**Figure 2:**
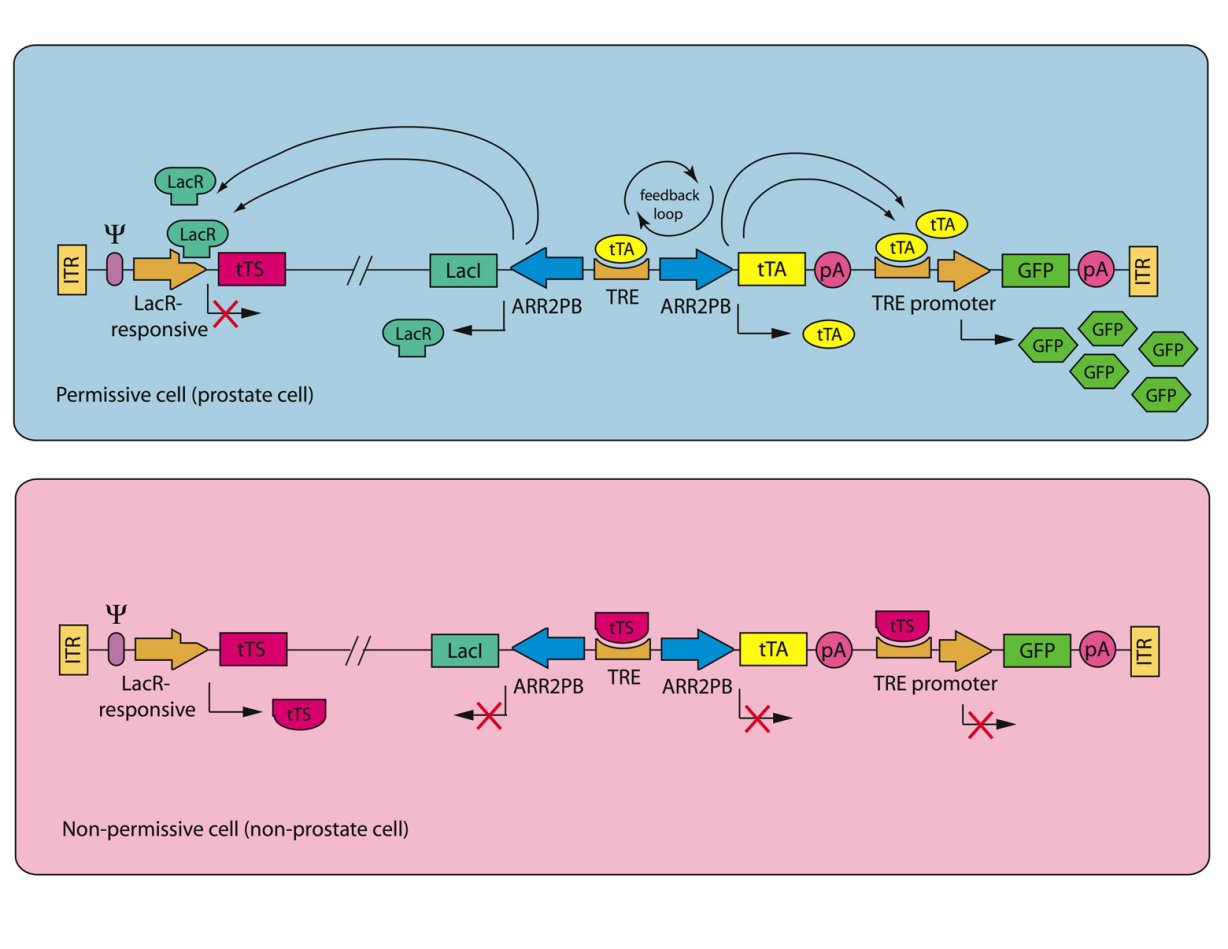
**Mechanism of action of the Ad/GFP_DiSTRES _vector**. *Mechanism of action of the Ad/GFP*_DiSTRES _*vector when transduced into a prostate cell (top panel)*. We hypothesize that the following sequence of events would occur in the prostate cell: (1) Both the *tTA *and the *LacI *genes would be induced, as a result of activation of the prostate specific TRE-ARR2PB promoters that drive the expressions of these genes; (2) Expression of tTA would then lead to transcriptional activation of the gene of interest (*e.g*., GFP), through binding of tTA to the TRE promoter; (3) The tTA protein would also bind to the TRE within its own TRE-ARR2PB promoter, thereby activating further transcription of itself and therefore establishing a positive feedback amplification loop of tTA expression; (4) Meanwhile, the LacR protein would bind to the LacR-responsive promoter of the *tTS *gene, thereby leading to suppression of *tTS *gene transcription; and (5) Finally, the tTA-driven amplification loop would lead to further expression of both the gene of interest and the LacR (and subsequently further repress the tTS). *Mechanism of action in non-prostate cells (bottom panel)*. In the non-prostate cell, we propose the following scenario: (1) Because the TRE-ARR2PB promoter is prostate-specific, neither the tTA nor the LacR proteins would be expressed; (2) In the absence of the LacR protein, the LacR-responsive promoter acts essentially like its constitutively active parental promoter (the CMV promoter). As a result, the *tTS *gene is now actively transcribed; and (3) Expression of the tTS protein would then lead to the transcriptional silencing of all TRE-regulated promoters, and therefore, the gene of interest (GFP), tTA and LacR would all be suppressed upon binding of the tTS to the TRE's within their respective promoters. *Note*: Tetracycline and/or doxycycline are not added to this system. Adding these drugs would act to negate the effects of the differential regulation of tTA versus tTS. The result would be basal expression of GFP in both cell types, and therefore, a loss in prostate-specificity.

In order to amplify prostate-specific expression further, we developed the novel DiSTRES regulation system. In this system we added two transcriptional silencer proteins, the tTS protein and the Lac repressor (LacR) protein, to the positive feedback loop design in order to differentially suppress gene expression depending upon the cell type transduced. The conceptual difference between PFLPS and DiSTRES is that in DiSTRES, the gene of interest is driven by the original TRE promoter instead of the prostate-specific ARR2PB-TRE promoter. From our previous experience with PS/TR, we found that the original TRE promoter often has some low level activity even in the absence of tTA expression. To counteract this activity, we added the tTS suppressor to decrease background transgene activation in non-target cells. However, in the setting of a prostate cell, tTS is not induced due to negative regulation from the LacR repressor (see Figs 1 and 2).

The design of the DiSTRES regulation system as cloned into an Ad5 vector deleted in E1, E3, and E4 (except for E4orf6) is depicted in Fig. 1. Control Ad vectors include Ad/*GFP*_TET_, which expresses GFP under the control of the traditional tet-off regulatory system, Ad/CMV-*GFP*, which expresses GFP under the control of the strong, constitutively active CMV promoter, and Ad/C.*LacZ*, which expresses β-galactosidase under the control of the CMV promoter. The designs of our earlier generation PS/TR and PFLPS vectors are also shown.

In the DiSTRES vector, three transgene cassettes were cloned into the right-end of the genome, which includes the following: (1) TRE-ARR2PB promoter driving Lac repressor (*LacI*) gene expression, cloned in reverse orientation, (2) ARR2PB promoter driving *tTA *expression, cloned just downstream of the TRE-ARR2PB.*LacI *cassette such that the TRE has bi-directional activity on both the *LacI *and *tTA *genes, and (3) original TRE promoter controlling GFP expression, cloned downstream of the ARR2PB.*tTA *cassette (see Fig. 1). In addition, a transgene cassette containing a newly developed LacR-responsive promoter (see Methods) was cloned into the left-end of the genome and controlled *tTS *gene expression (see Fig. 1).

The cloning capacity of our E1/E3/E4(except orf6)-deleted Ad vector is approximately 7.5 kb. Therefore, several strategic cloning measures had to be taken in order to conserve sufficient space to include all four transgene cassettes into a single complex adenovirus vector (see Methods). In addition, special consideration was made in the construction of this complex rAd vector based upon our previous findings regarding interference from the E1a enhancer. Because we previously had found that the basal activity of both the TRE and the ARR2PB promoters were significantly affected by interference from the E1a enhancer [10], we designed the Ad/*GFP*_DiSTRES _vector so that all TRE and TRE-ARR2PB promoters were away from the E1 region by placing them near the right ITR (Fig. 1). Meanwhile, the transgene cassette containing the LacR-responsive promoter driving tTS expression was cloned near the left ITR (Fig. 1). The theoretical mechanism of action of the DiSTRES regulation system is diagrammed in Fig. 2.

### Levels of prostate-specific expression from Ad-delivered DiSTRES are greater than that induced by either the CMV promoter or tet-regulated controls

In order to determine the prostate-specificity of the Ad/*GFP*_DiSTRES _vector, two prostate cancer cell lines, LNCaP and PC3/AR, and two non-prostate cell lines, HeLa and U251MG, were chosen for *in vitro *analysis. Since the ARR2PB promoter is known to be inducible by androgen (*e.g*., dihydrotestosterone; DHT) [7], the LNCaP cell line was chosen specifically because it is one of the few prostate cancer cell lines available that express functional androgen receptor (AR). The PC-3 cell line is known to be AR negative, and therefore, an AR stable transfectant PC-3 cell line (PC3/AR) was chosen [11] for the Ad/*GFP*_DiSTRES _characterization studies. In order to directly compare vector activity in prostate versus non-prostate cells, all cell types were infected in the presence of DHT, even though DHT would not likely be present under physiological tissues.

**Figure 3:**
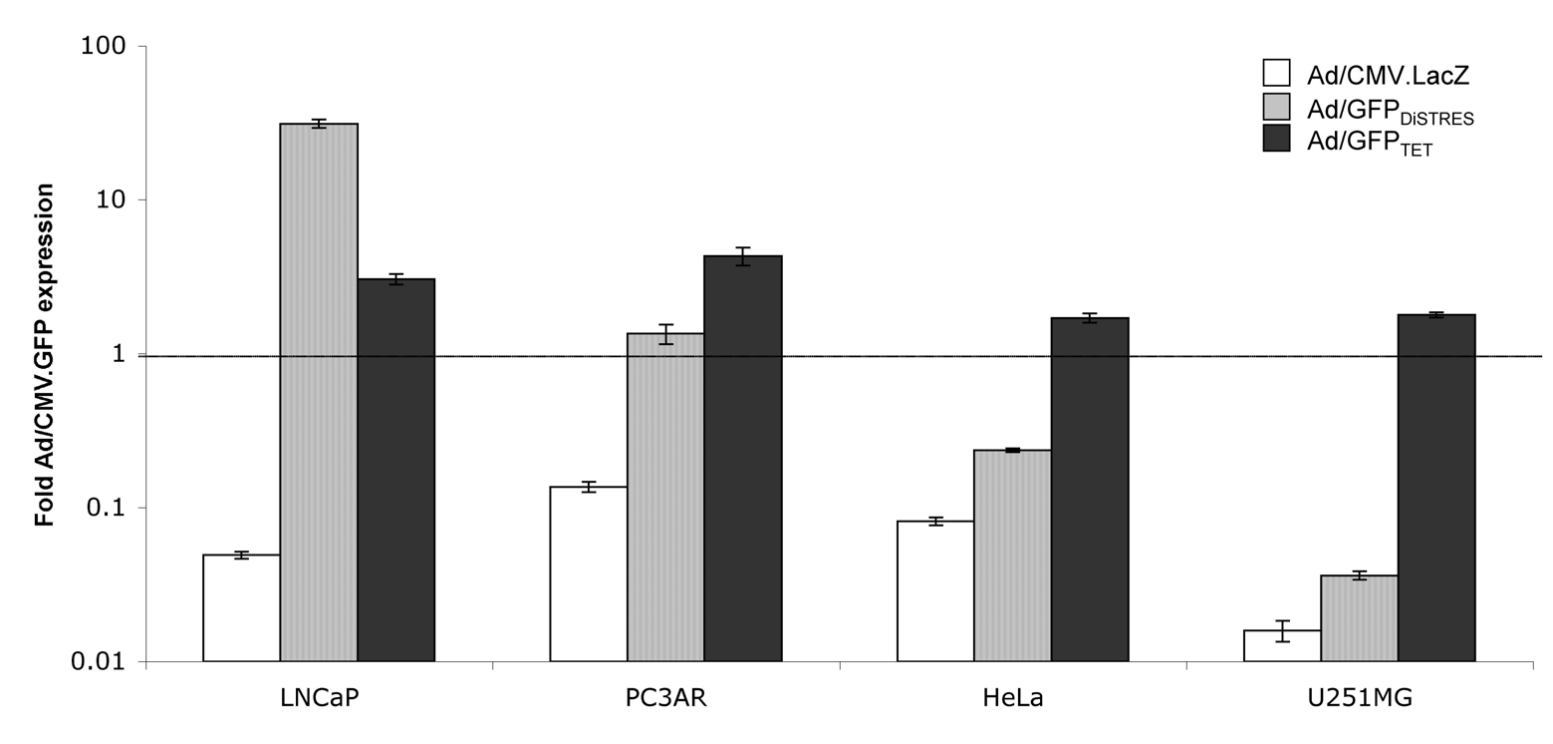
**Comparison of DiSTRES- and CMV-mediated gene expression**. LNCaP, PC3AR, HeLa and U251MG cells were seeded at 1.25 × 10^4 ^cells/well in 96-well plates and infected with complex Ad vectors at MOI 30, 200, 200, and 50, respectively, in the presence 30 nM DHT. 2 days post-infection, cell lysates were assayed for GFP fluorescence. Infection with Ad/C.LacZ served as a negative control infection. Results are displayed as fold expression, setting GFP fluorescence from Ad/CMV.*GFP *infection at 1 for each cell line. Due to differing transduction efficiencies among cell lines, each cell type was infected at an optimized MOI, based on pilot dose response studies conducted with the Ad/*GFP*_TET _vector. MOI values were based on IU/ml. *p < 0.05 compared to Ad/CMV.*GFP*. †p < 0.05 compared to Ad/GFP_TET_.

For *in vitro *analysis, both prostate and non-prostate cell lines were infected with Ad/*GFP*_DiSTRES _vector and controls (Fig. 3). Due to differing transduction efficiencies among cell lines, each cell type was infected at an optimized MOI, based on pilot dose response studies conducted with the Ad/*GFP*_TET _vector. Data are represented as fold GFP expression, setting infection with the Ad/CMV-*GFP *control vector at 1. As demonstrated in Fig. 3, Ad/*GFP*_DiSTRES _vector induced high levels of GFP expression in the prostate cancer cell lines without a loss in prostate specificity. In PC3/AR cells, the Ad/*GFP*_DiSTRES _vector induced GFP expression to levels similar to the non-specific, constitutively active Ad/CMV-*GFP *control. Remarkably, GFP induction by the Ad/*GFP*_DiSTRES _vector in LNCaP cells was not only significantly higher than that seen with Ad/CMV-*GFP *(p = 0.043) but also significantly amplified, even in comparison to the Ad/*GFP*_TET _vector (p = 0.038). In fact, the activity from Ad/*GFP*_DiSTRES _was over 30-fold higher than Ad/CMV-*GFP *and over 10-fold higher than the highly induced tet-regulated Ad/*GFP*_TET _vector, when transduced in LNCaP cells. Meanwhile, the levels of GFP expression in the HeLa and U251MG cells were similar to background, with a low level of GFP expression seen in HeLa cells.

### The Ad/*GFP*_DiSTRES _vector demonstrates improved prostate-specific GFP induction compared to the earlier generation, Ad/*GFP*_PS/TR _and Ad/*GFP*_PFLPS _vectors

**Figure 4:**
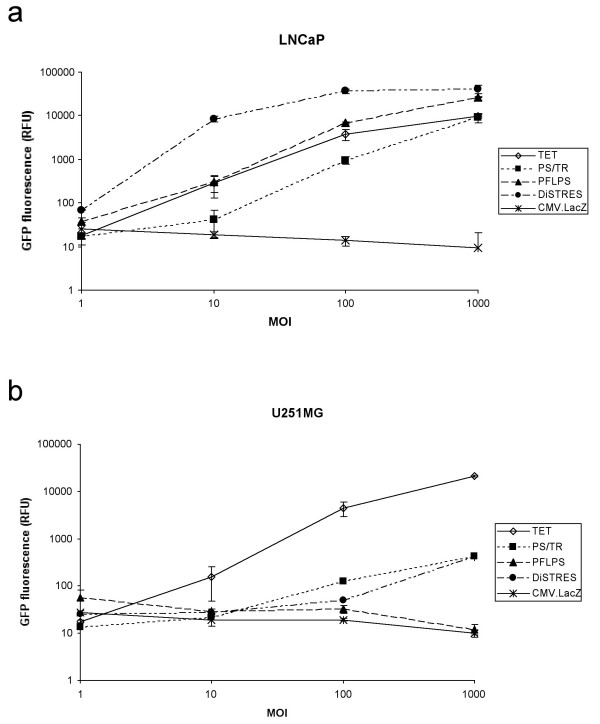
**Ad/GFP_DiSTRES _demonstrates specificity for prostate cells, decreased activity in non-prostate control cells, and improves upon earlier vector designs**. GFP fluorescence of *(a) *LNCaP cells and *(b) *U251MG cells. LNCaP and U251MG cells were seeded at 1.25 × 10^4 ^cells/well of a 96-well plate and infected with Ad/GFP_TET_, Ad/GFP_PS/TR_, Ad/GFP_PFLPS_, Ad/GFP_DiSTRES_, or Ad/C.LacZ (negative control) in the presence of 30 nM DHT at MOI 1, 10, 100, or 1000. 2 days post-infection, cell lysates were assayed for GFP fluorescence. RFU: relative fluorescence units. MOI values were based on IU/ml.

We wanted to determine whether the DiSTRES system had improved upon our earlier generation designs, the PS/TR and PFLPS systems. In order to conduct a comparative analysis of these three prostate-specific regulation systems, we infected LNCaP and U251MG cells in parallel with Ad/GFP_PS/TR_, Ad/GFP_PFLPS_, Ad/GFP_DiSTRES_, Ad/GFP_TET_, or Ad/C.LacZ and assayed for GFP expression (Fig. 4). As demonstrated in Fig. 4, the activity of the Ad/GFP_PS/TR _vector (which lacks the positive feedback loop) was less than that induced by either Ad/GFP_PFLPS _or Ad/GFP_DiSTRES _in LNCaP cells at all MOI's tested. Overall, the Ad/GFP_DiSTRES _vector demonstrated the highest levels of prostate-specific GFP expression at all MOI's tested. In addition, the Ad/GFP_PS/TR _vector demonstrated more non-specific expression in U251MG than either Ad/GFP_PFLPS _or Ad/GFP_DiSTRES_, except at MOI 1000, where the Ad/GFP_DiSTRES _vector loses some of its specificity. Finally, Fig. 4 visually demonstrates the difference in GFP induction and specificity of Ad/GFP_DiSTRES _versus Ad/GFP_TET _in LNCaP and U251MG cells at MOI 100.

### DiSTRES-mediated FasL-GFP cytotoxicity is prostate-specific

**Figure 5:**
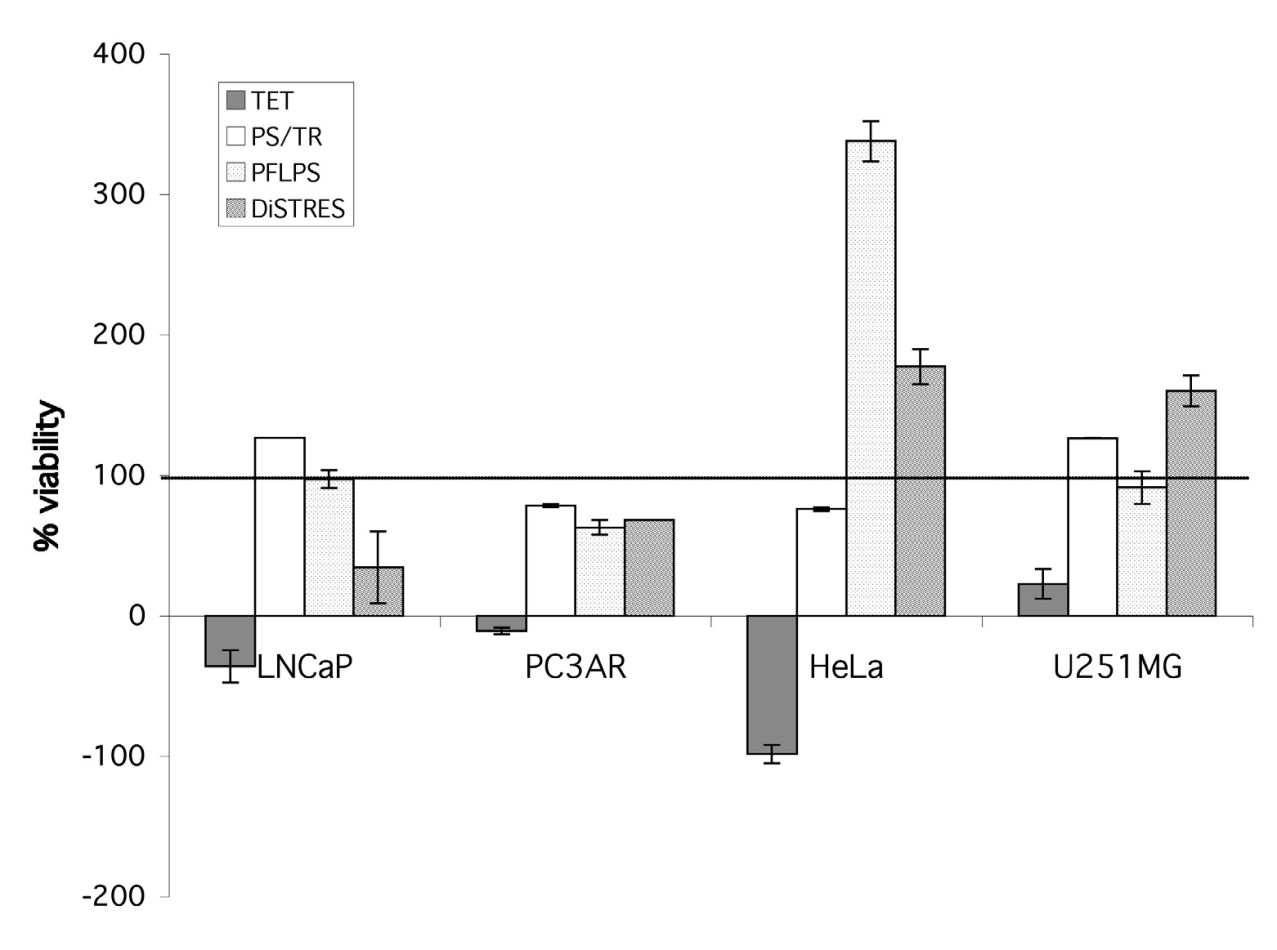
**Ad/FasL-GFP_DiSTRES _demonstrates preferential cytotoxicity in prostate cells**. LNCaP, PC3AR, U251MG, and HeLa cells were seeded at 1.25 × 10^4 ^cells/well in a 96-well plate and infected with Ad/FasL-GFP_TET_, Ad/FasL-GFP_PS/TR_, Ad/FasL-GFP_PFLPS_, Ad/FasL-GFP_DiSTRES_, or 1× HBS (mock) in the presence of 30 nM DHT. LNCaP, PC3AR, U251MG, and HeLa were infected at MOI 10, 5, 5, and 2.5, respectively. Three days post-infection, cells were fixed with methanol and then assayed for cytotoxicity using crystal violet assay. Since each cell type showed varying sensitivities to adenovirus-delivered FasL-GFP, we infected each cell type at MOI values determined during pilot studies to induce maximal cytotoxicity in response to Ad/*FasL-GFP*_TET _control. Percent viability was calculated by dividing the mean absorbance of each sample by the mean absorbance of 1× HBS mock infection for each cell line and then multiplying by 100%. MOI values were based on pfu/ml. Horizontal line delineates 100% viability.

In order to characterize the DiSTRES regulation system in the context of a prostate cancer therapeutic, we first subcloned the *FasL-GFP *fusion gene in the place of the *GFP *gene within Ad/*GFP*_DiSTRES_, resulting in the Ad/*FasL-GFP*_DiSTRES _vector. We then infected the same panel of prostate and non-prostate cell lines described in Fig. 3 with Ad/*FasL-GFP*_DiSTRES _vector and controls and assayed for resultant cell viability (see Fig. 5). Since each cell type showed varying sensitivities to adenovirus-delivered FasL-GFP, we infected each cell type at MOI values determined during pilot studies to induce maximal cytotoxicity in response to Ad/*FasL-GFP*_TET _control.

The cytotoxicity of all of the prostate-specific vectors was prostate-specific, as there was increased cell viability in the U251MG and HeLa cells when compared to the Ad/*FasL-GFP*_TET _control. In LNCaP, cell viability in Ad/*FasL-GFP*_DiSTRES_-transduced cells was greatly reduced although not to the same degree as our positive control for apoptosis, Ad/*FasL-GFP*_TET_. In PC3AR, similar levels of cell viability were seen among the three prostate-specific vectors.

**Figure 6:**
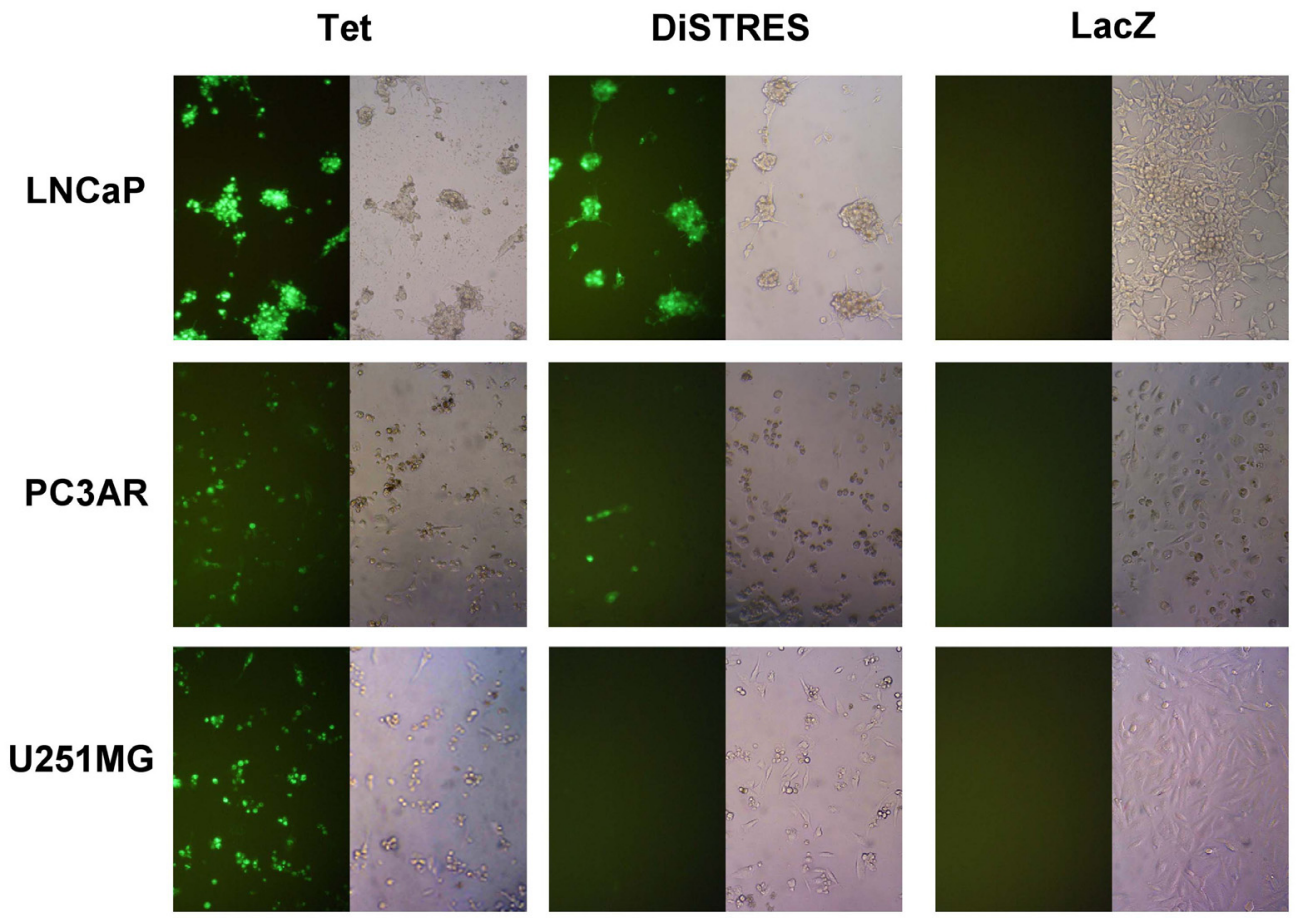
**Ad/FasL-GFP_DiSTRES _demonstrates prostate-specific cytotoxicity as indicated by rounded morphology of prostate cancer cells**. LNCaP, PC3AR, and U251MG cells were seeded at and infected as described in Fig. 5. LNCaP, PC3AR, and U251MG were infected at MOI 10, 5, and 5, respectively. Two days post-infection, cells were visualized using Axiovert-25 fluorescent microscope with FITC filter set and photos taken with Pixera digital camera at 100× magnification. Photos on the left were taken under UV light. Photos on the right were taken under bright field setting. MOI values are based on pfu/ml.

In comparison to the PS/TR and PFLPS vectors, the DiSTRES vector demonstrated the highest level of cytotoxicity in LNCaP cells. However, the cytotoxic activity of Ad/*FasL-GFP*_DiSTRES _compared to mock infection was not statistically significant. Although the cell viability studies did not reveal a statistical difference between Ad/*FasL-GFP*_DiSTRES _and control, both prostate cancer cell types did demonstrate the typical cell-rounding morphology as a result of Ad/*FasL-GFP*_DiSTRES _infection (Fig. 6). However, U251MG seemed to be somewhat affected by Ad/*FasL-GFP*_DiSTRES _infection, as well.

## Discussion

A wide range of gene therapy vector approaches is being investigated as potential cancer therapeutics including manipulation of cell cycle control, apoptosis strategies, suicide gene therapy, tumor-selective replicating virus vectors, angiogenesis therapy and immunotherapy. Recent successes in cancer gene therapy include the approvals of the Ad/p53 vector known as Gendicine from SiBiono GeneTech Co. and the oncolytic Ad vector known as H101 from Sunway Biotech Co. for clinical use in China [3,4]. The activity of both of these adenovirus-based vectors takes advantage of the fact that over 50% of all cancers have mutations in p53. Additionally, evidence from Onyx's ONYX-015 and Sunway's H101 vectors appear to indicate that these oncolytic vectors also have activity in non-p53 mutant cancer cells as well [3].

When designing gene therapy vectors for the treatment of cancer, along with selection of therapeutic transgene, an equally important aspect to take under consideration is how to target gene expression. This is particularly important considering that the two initial phases of clinical characterization (Phase I and II clinical trials) focus on safety. The cancer gene therapy vector most likely to demonstrate a significant clinical result will be one that initiates a large therapeutic index (*i.e*., high potency against malignant cells), while keeping toxicity to normal tissues at a minimum. The use of an amplified feedback loop strategy to tightly regulate yet highly induce gene expression may be key to the development of additional cancer therapeutic vectors.

In this study, we have constructed and characterized a novel transcriptional regulation system that incorporates both an amplification feedback loop, as well as two tissue-selective repressor elements, which differentially suppress specific transcriptional events, depending upon the cell type transduced. This differentially suppressible tetracycline regulated expression system (DiSTRES) demonstrated prostate-specific transgene induction, as the Ad/GFP_DiSTRES _vector was found to induce GFP expression levels in PC3/AR cells to levels similar to that of the CMV promoter, a promoter which is well-recognized as a strong, constitutively active promoter of non-specific gene expression. Moreover, the Ad/GFP_DiSTRES _vector induced GFP expression levels in LNCaP cells to levels that significantly exceeded those of both the CMV promoter as well as the tet-regulated system. This induction by Ad/GFP_DiSTRES _was approximately 30 times the activity of Ad/CMV-*GFP *and approximately 10 times the activity of Ad/GFP_TET_. Additionally, the Ad/GFP_DiSTRES _vector retained specificity in the two non-prostate cell line controls, HeLa and U251MG. Levels of GFP expression from the Ad/GFP_DiSTRES _vector, upon transduction of these non-prostate cell lines, were similar or slightly higher than background.

Finally, although LNCaP cells are known to be resistant to FasL-mediated apoptosis, the Ad/FasL-GFP_DiSTRES _vector demonstrated cytotoxic effects in LNCaP and somewhat modest effects in PC3AR cells. This overcoming of FasL resistance was seen previously with our tet-regulated Ad/FasL-GFP_TET _vector [12], and we believe it is due to an overexpression of FasL-GFP overcoming a certain threshold in these resistant cells. Once the cells are saturated with FasL-GFP, the cell then appears to become susceptible to the FasL-mediated apoptosis.

A two-step transcriptional amplification (TSTA) system has been described which utilizes a Gal4-VP16 fusion protein instead of tTA for amplification of gene expression [13-17] and is similar to our previous PS/TR system [9]. In transfection experiments, Zhang et al. demonstrates a 10-fold increase in prostate-specific luciferase expression in LNCaP, compared to CMV [17]. In another report, Block et al. claims an amplification of up to 590-fold of luciferase expression in SKCO1 cells compared to CMV [13]. Similar results have not been repeated by other groups using the TSTA system. The specificity of Block et al's vector, however, was greatly compromised as a result. In their negative control cell line, HepG2, their Ad.Muc_bin_-luc vector demonstrated 7-fold higher gene expression than CMV at MOI 1. The authors do not explain the reasoning for this very high background activity in non-target cells [13]. Such increased expression in non-target cells could potentially be mitigated utilizing a system similar to DiSTRES. However, it is difficult to compare the two systems since many papers do not describe the activity of the TSTA in non-target cells. Further study will need to be conducted to truly compare the two systems.

The DiSTRES transcriptional regulation system has three characteristics that separate it from other regulation systems published in the literature: (1) differential expression of two transcriptional repressor proteins (tTS or LacR) depending on the cell type transduced; (2) incorporation of a positive feedback amplification loop of prostate specific gene expression; and (3) the cloning of the entire system into a single complex recombinant Ad vector, thus preventing the need for co-infection with two or more separate Ad vectors. In fact, most Ad vectors published in the literature (gutless Ad vectors included) contain only a single transgene cassette. This, therefore, makes the progressiveness of the Ad/GFP_DiSTRES _and Ad/FasL-GFP_DiSTRES _vectors rather uncommon as both of these vectors each contained four expression cassettes.

Application of the DiSTRES regulation system is not restricted to only the gene therapy treatment of prostate cancers. Its complex transcriptional regulation concept could be transferred to other tissue types by simply incorporating different tissue-specific promoters, thereby expanding its potential utility to include gene therapy of other cancers and molecular genetic applications other than cancer, such as the gene therapy treatment of genetic disorders, development of gene-based vaccines expressing immunogenic bacterial or viral antigens, and development of new transgenic mouse models that require highly induced, organ-restricted expression of a particular gene of interest. Finally, the effective transcriptional regulation afforded by DiSTRES could be combined with current methods of transductional regulation including manipulation of Ad fiber knob [18-24] and use of bi-specific antibodies [25-29] to further improve the targeting of gene therapy vectors to specific cell types and therefore increase the specificity and the safety of the vectors.

## Conclusion

We have developed and characterized a novel transcriptional regulatory system that demonstrates highly induced, prostate-specific expression that retains its tissue-specificity. Such a regulation system could be potentially modified to include tissue- or cancer-specific promoters in the place of the ARR2PB promoter as well as any desired therapeutic transgene and thus is ideal for several molecular genetic and gene therapeutic applications that require highly induced, organ-restricted expression of a particular gene of interest. This transcriptional regulation can also be combined with transductional regulation systems to increase the specificity and the safety of current gene therapy vectors. The DiSTRES regulation system, therefore, serves as a promising new approach for the transcriptional regulation of therapeutic genes for future molecular genetic and therapeutic applications.

## Methods

### Cell lines

HEK293 (human embryonic kidney) and LNCaP (prostate cancer) cells were obtained from American Type Culture Collection (ATCC; Manassas, VA). U343MG and U251MG (brain tumor) cell lines were obtained from the Brain Tumor Research Center Tissue Bank (Dept. of Neurological Surgery, UCSF, San Francisco, CA). PC3/AR (the human prostate cancer cell line, PC-3, stably transfected with human androgen receptor cDNA) was generously provided by Kerry L. Burnstein. All cell lines were maintained in media supplemented with 10% cosmic calf serum (CCS; HyClone, Logan, UT), with HEK293 being maintained in DMEM, LNCaP being maintained in RPMI, PC3/AR being maintained in RPMI containing 350 μg/ml active G418, and U343MG and U251MG being maintained in MEM.

### Construction of plasmids vectors

The pUHD 10-3 (containing the TRE promoter), the pUHD 15-1 (containing the *tTA *gene), and the pUHS 6-1 (containing the *tTS *gene) plasmids were generously provided by Hermann Bujard. The ARR2PB (0.45 kb) promoter was developed in the laboratory of Robert J. Matusik, who contributed the pARR2PB.PolI.TRZ-SK vector. Construction of pLAd-CMV, pLAd-mcs and pRAd-T.*GFP *vectors has been described previously [12].

For construction of the DiSTRES vectors, first, the *tTA *gene and BGH polyA were excised as a single fragment from pUHD 10-3.B (a modified version of pUHD 10-3 which includes a polyA from BGH in place of the original SV40 polyA) and subcloned in place of the SEAP gene within the pLAd(2Pb.SP)_r _plasmid (described previously) [10], resulting in the pLAd(2Pb.*tTA*.B)_r _vector. Next, the ARR2PB.*tTA*.B cassette from pLAd(2Pb.*tTA*.B)_r _was subcloned upstream of the TRE.*GFP *cassette of pRAd^2^T.*GFP*.B to obtain the pRAd^2^(2Pb.*tTA*.B)B(T.*GFP*.B) plasmid. Next, the *LacI *gene was PCR amplified from the pCMVLacI plasmid (Stratagene) and then subcloned in the place of the *GFP *gene within the pRAd^2^T2Pb.*GFP*.B plasmid (described previously) [5], resulting in the pRAd^2^T2Pb.*LacI*.B plasmid. Finally, the T2Pb.*LacI *cassette was subcloned in reverse orientation upstream of the ARR2PB.*tTA *cassette within pRAd^2^(2Pb.*tTA*.B)B(T.*GFP*.B), leading to construction of the pRAd^2^(T2Pb.*LacI*)_r_(2Pb.*tTA*.B)B(T.*GFP*.B) right-end shuttle plasmid of DiSTRES. By placing the T2Pb.*LacI *cassette in reverse and upstream of he ARR2PB.*tTA *cassette, this resulted in both the *LacI *and the *tTA *genes sharing a TRE, thus conserving on much needed space, as well as placing both genes under the control of TRE-ARR2PB promoters.

The LacR-responsive promoter was developed by PCR-amplifying the CMV promoter using oligos containing lac operator (o_i_) sequences to modify the CMV promoter. These oligos were designed to introduce a single lac operator near the 5' end of CMV as well as two lac operators flanking the TATA box near the 3' end of the promoter. The oligo sequences, 5'-TTTTTTTTTACTAGTGGAATTGTGAGCG CTCACAATTCCACATTAATAGTAATCAATTACGGGGTCATTAG-3' forward; and 5'-TTTTTTTTTGGATCCTGTGGAATTGTGAGCGCTCACAATTCCACATCGAAATTCCGCGGACCGGTCGCCACCATGGTGAGCAAGGGCGAGGA-3' reverse, were used to construct the LacR-responsive promoter, using pcDNA3.1 (Invitrogen) as a template. The LacR-responsive promoter PCR fragment was then cloned in the place of the CMV promoter within the pUHS 6-1 plasmid to yield the pUHS Co_i_2.*tTS*.S plasmid. Finally, the Co_i_2.*tTS *cassette was subcloned into the pLAd-CMV plasmid in place of the CMV promoter, resulting in the pLAd.Co_i_2.*tTS *left-end Ad shuttle plasmid vector.

It should be noted that the cloning capacity of our E1/E3/E4(except orf6)-deleted Ad5 vector is approximately 7.5 kb and that the total size of the DiSTRES regulation system just fits or slightly exceeds this value (~7.4 kb with GFP; ~8.16 kb with FasL-GFP). Therefore, in order to conserve on space, both the *tTS *and the *LacI *genes were cloned into their respective shuttle plasmids without polyadenylation signals. PolyA sites were not required for these two particular transgene cassettes due to their orientations in relation to the deleted Ad genes within the Ad vector genome. The polyA sites for the E1b and E4 genes can be utilized for the polyadenylation of the *tTS *and the *LacI *genes, respectively.

### Construction of recombinant adenoviral vectors

Construction of Ad/C.*LacZ *and Ad/*GFP*_TET _has been described previously [12]. The pRAd^2^(T2Pb.*LacI*)_r_(2Pb.*tTA*.B)B (T.*GFP*.B) plasmid was digested with *Swa*I and *Spe*I while the pLAd.Co_i_2.*tTS *plasmid was digested with *Swa*I and *Avr*II. The digested pLAd.Co_i_2.*tTS *and pRAd^2^(T2Pb.*LacI*)_r_(2Pb.*tTA*.B)B(T.*GFP*.B) fragments were gel-purified and then ligated to a gel-purified Ad5 genome backbone (Ad5sub360SR) digested on both ends with *Xba*I. All Ad vectors are based on Ad5sub360SR, which contains deletions in E3 and all E4 ORFs with the exception of ORF6. The Ad/*FasL-GFP*_DiSTRES _vector genome was constructed in the same way as described for Ad/*GFP*_DiSTRES_, using a pRAd shuttle vector containing FasL-GFP subcloned in the place of GFP.

### Propagation of recombinant adenovirus vectors

All vectors were propagated in HEK293 cells, using standard procedures [12,30,31]. Briefly, HEK293 cells were transfected with the complex Ad vector genome ligation mixture using Fugene 6 transfection reagent (Roche, Indianapolis, IN) following manufacturer's instructions. Transfected cells were maintained until adenovirus-related cytopathic effects (CPE) were observed (typically 7–14 days post-transfection), at which point the cells were collected. Vector propagation and amplification was then achieved by standard techniques. Briefly, adenoviral lysates from twenty-four 150 mm plates were banded twice on CsCl gradients and desalted twice with a PD-10 size exclusion column (Amersham Scientific, Piscataway, NJ) into HEPES buffered saline (HBS; 21 mM HEPES, 140 mM NaCl, 5 mM KCl, 0.75 mM Na_2_HPO_4_·2H_2_O, and 0.1% (w/v) dextrose; adjust pH with NaOH to 7.5; and filter sterilize) containing 5% glycerol, and stored at -70°C.

### Titering of recombinant adenovirus vector by infectious units

All vectors were titrated on HEK293 cells infected in serial dilution on triplicate columns of 96-well plates for either GFP fluorescence or X-gal staining. GFP fluorescence was monitored with Axiovert-25 fluorescent microscope (Carl Zeiss, Germany) and FITC excitation/emission filter set (Chroma Technology Corp, Rockingham, VT) two days post-infection. Cells infected with Ad/C.*LacZ *were fixed two days post-infection with fixative solution (2% formaldehyde, 0.05% glutaraldehyde in 1× PBS) for 5 minutes at room temperature and then stained overnight at 37°C in X-gal solution (1 mg/ml X-gal (5-Bromo-4-chloro-3-indolyl-β-D-galactopyranoside), 5 mM potassium ferricyanide, 5 mM potassium ferrocyanide, 2 mM MgCl_2 _in 1× PBS). The resulting titers were scored as infectious units (IU) per ml.

### Titering of recombinant adenovirus vector by particle forming units

All vectors were titrated on HEK293 cells infected in serial dilution on duplicate wells of 12-well plates. The day prior to infection, HEK293 cells were seeded with 2 × 10^5 ^cells/ml seeding density at 1 ml/well. 4 to 16 hrs post-infection, cells were overlaid with 1 ml/well of 0.4% agar (w/v) in DMEM/2% CCS and incubated at 37°C for 5–7 days until indications of CPE were observed. Cells were fixed with 7% formaldehyde (v/v in dH_2_O) solution at room temperature for 1 hr. Formaldehyde was aspirated from wells, and agar plugs were carefully removed. Fixed cells were stained for 10 min at room temperature with 0.2% crystal violet (w/v in dH_2_O). Plaques were visualized using a standard light box. The resulting titers were scored as plaque forming units (pfu) per ml. In comparison to IU titers, pfu titers were approximately 2 logs lower. For example, the Ad/C.LacZ vector titer was 2.02 × 10^11 ^IU/ml in infectious units and was 2.40 × 10^9 ^pfu/ml in particle-forming units.

### *In vitro *infections

1.25 × 10^4 ^cells/well were seeded in 96-well plates. Seeded cells were infected 3 hours post-seeding at varying multiplicities of infections (MOI), depending on the cell line. MOI calculations were based on cell numbers at the time of seeding and on Ad vector titers based on IU/ml and pfu/ml, for GFP infections and FasL-GFP infections, respectively. In order to directly compare vector activity in prostate versus non-prostate cells, all cell types were infected in the presence of DHT, even though DHT would not likely be present under physiological conditions in non-prostate tissues.

### Quantification of GFP expression

GFP fluorescence in cells was visualized 48 hours post-transduction using Axiovert-25 fluorescent microscope with FITC filter set. For quantitative analysis of GFP activity, cells were lysed with 0.5% Triton x-100 in 1× PBS. Cell lysates were transferred to 96-well black microtiter plates (BMG Labtechnologies, Offenburg, Germany) and relative GFP fluorescence was measured using FLUOstar™ dual fluorescence/absorbance plate reader (BMG Labtechnologies) at excitation 485 nm and emission 520 nm.

### Quantification of cell viability

Two or three days post-infection, cells were fixed in 100% methanol overnight at -20°C. Cell monolayers were stained with 0.2% crystal violet for 2 min at room temperature. Plates were washed twice with dH_2_O and then destained with 50 μl/well of destain solution (30% methanol, 10% acetic acid in dH_2_O) for wells of 96-well plate. Plates were assayed for absorbance at 620 nm. Percent viability was calculated by dividing the mean absorbance of each sample by the mean absorbance of 1× HBS mock infection and then multiplying by 100%.

### Statistical analysis

All assays were performed in duplicate and the results were expressed as the mean ± standard error of the mean (SEM). Statistical analysis was determined using a two-sample t-test.

## Authors' contributions

JW constructed the DiSTRES adenovirus vectors, conducted all characterization experiments on the constructs, and also drafted the manuscript. SR participated in the overall design and cloning strategy for the DiSTRES constructs. HY constructed the control vectors and participated in the construction of the DiSTRES adenoviral constructs. JY conceived of the study and participated in the overall vector designs. All authors read and approved the final manuscript.
